# Scaling analysis and experiments for the apparent stiffness of woodpile lattices with tubular struts

**DOI:** 10.1038/s41598-025-23890-3

**Published:** 2025-11-17

**Authors:** Faezeh Shalchy, Sina Askarinejad, Atul Bhaskar

**Affiliations:** 1 School of Engineering and Physical Sciences, Heriot-Watt University, Edinburgh, EH14 4AS UK; 2 School of Science and Engineering, University of Dundee, Dundee, DD1 4HN UK; 3 Faculty of Engineering and Physical Sciences, University of Southampton, Southampton, SO17 1BJ UK

**Keywords:** Engineering, Materials science

## Abstract

The apparent stiffness of woodpile lattices with tubular struts compressed diametrically in the stacking direction is investigated theoretically, experimentally and computationally. This architecture is inspired by multifunctional application in tissue engineering, where hierarchical porosity is important. A key result presented here is the absence of a simple power law of the form $\langle E\rangle \sim (\overline{\rho })^{m}$ relating the apparent modulus to the apparent density, where *m* is an exponent. Here we show that the relationship $\langle E \rangle (r/t)^{\frac{1}{2}} \sim (\overline{\rho })^{2}$ holds between the apparent modulus of elasticity and the geometric and materials parameters. This structure-property relationship is further examined computationally and experimentally. We successfully fabricated these structures using affordable commercial 3D printing machines that allow us to control properties by adjusting tube dimensions, thus offering new possibilities for optimizing structural performance. Compression tests confirm that replacing solid struts with tubular ones significantly reduces stiffness—up to an order of magnitude—at equivalent porosity levels. This reduction in stiffness underscores the crucial role of tubular strut geometry in determining the mechanical response of engineered lattices. Our findings provide valuable insights into the design and manufacture of advanced lattice structures, paving the way for customisable multifunctional materials.

## Introduction

Lattices with tubular struts have gained significant interest due to their potential for lightweight structures, energy absorption, and mechanical tunability. The present work is motivated by applications in tissue engineering where biomedical scaffolds require hierarchical porosity to facilitate cell adhesion, proliferation and growth. These hollow elements not only reduce weight and provide flexibility, they also enhance functionality by improving mechanical energy dissipation and enabling fluid and nutrient transport, in addition to providing space for cells to grow within tissue engineering scaffolds. Their unique geometry allows us to tailor mechanical properties, making them highly versatile for biomedical engineering and industrial applications. An architected material utilises a combination of several simple materials structured in a way that the resulting material outperforms its constituents due to careful engineering of the internal structure. Lattices with various geometrical shapes have been manufactured and their mechanics have been studied under different loading conditions^1–3^. Hexagonal honeycomb, tetrahedral, pyramidal, 3D-kagome, diamond, octahedral and egg box are among those^4–7^. Additive manufacture has enabled the invention of a wide range of single and multi-phase lattice materials, including square lattice or woodpile lattices^8^. Lattices fabricated in the form of a woodpile using additive manufacturing are some of the most common structures for biomedical applications^9–11^. Long slender structures are joined in various connectivity to form repetitive structures, here we will designate these building blocks as *s*truts.

Mechanical response of additively manufactured lattice materials and structures is of great current interest, because the structural performance could be optimised as per the requirements of an application. The apparent stiffness of woodpile lattices with cylindrical solid struts depends on the geometric parameters such as spacing between struts, strut diameter, stacking arrangement, and the inevitable overlap between fused layers. Mechanics of such structures with *s*olid cylindrical building blocks under compression was studied before^12,13^. In woodpile lattices with tubular struts, tube thickness is an extra geometric parameter for tailoring the apparent properties, thus becoming a promising concept for realising *d*esigner materials^14^. It is well known that bending stiffness of a hollow tube is greater than that of a solid cylinder of the same cross-sectional area. The reason for this is the increased second moment of cross-sectional area, given a fixed amount of material; thus the potential of enhanced material stiffness per unit mass. Bending stiffness of struts can be increased by introducing porosity to the struts, however control on the stiffness and the structural efficiency is poor when the porosity is random, as in that achieved by foaming^15^. Therefore, a host of elastic meta materials with precisely tuned stiffness can be fabricated by stacking tubular struts in layers. The use of tubular or hollow struts substantially increases the resistance to filament buckling^16^, because for the same cross-sectional material, as tubes provide greater cross-sectional second moment of area. This increase in buckling resistance, has made hollow tubes a great candidate for sandwich panel cores^17,18^.

Hollow struts have been used within lattices, without the use of additive manufacturing^4,16^. Queheillalt et al.^17^ manufactured a sandwich panel consisting of a periodic diamond hole pattern sheet made of 304L stainless steel hollow tubes and two steel face sheets. The out-of-plane compression properties of this hollow truss lattice structure with density of 2.8 % was found to be approximately twice that of a similar lattice made of solid trusses. Dong et al.^19^ fabricated collinear lattice structures using 304 stainless steel hollow tubes and investigated the effect of tube wall thickness for square and diamond lattice topologies with relative densities between 0.03 and 0.11. Based on their mechanical characterisation of specimens, the compressive strength of lattices with hollow struts along the direction of struts is greater compared to the lattices with solid struts and same porosity level^19^. Despite these advantages, model generation and design of lattices with hollow struts are challenging due to the more complex shapes of structures. In all the previous studies, either (a) the concepts are theoretical without any physical realisation of the structures, or (b) the fabrication has involved painstaking process. Here we respond to these manufacturing challenges by providing an innovative solution to the fabrication of such structured materials using affordable 3D printers and bespoke dies. Additionally, we provide simple analytical formula for the apparent stiffness in a form readily usable by material designers.

Lattices with tubular struts are particularly important in tissue engineering applications. Biomedical scaffolds require a certain degree of porosity and mechanical integrity based on their application to ensure clinical success^20^. In particular, bone scaffolds need to possess enhanced mechanical and physical properties to be suitable as bone grafts^21^. In bone tissue engineering, hollow struts help the spread and transport of nutrition and oxygen, and further facilitate the new bone formation in the inner section of scaffolds. Although many 3D printed scaffolds possess high porosity for tissue regeneration, these macropores in the scaffolds cannot form channel structure effectively. Moreover, these pores can block the growth of rudimentary vasculature and even the interior new bone tissues^22^. Therefore, introducing hollow channels into the printed scaffolds could potentially be beneficial for bioengineering reasons. The advantage of tubular struts within tissue engineering scaffolds is shown through cell viability investigations on scaffolds with or without an embedded microfluidic channel^23–26^. Zhang et al.^27^ demonstrate the effectiveness of hollow channels in improving rabbit bone regeneration through enhanced blood vessel and new bone formation. Methods in the literature for manufacturing of vessel-like constructs and scaffolds containing tubular struts using 3D printing are cumbersome^12^ and sometimes not suitable for creating vessels within the scaffolds. As an example, foam scaffolds containing tubes were previously fabricated through coaxial dies^27,28^. The accomplishments to date are nothing short of remarkable. While all the developed techniques are very powerful and have the potential for a precise control of structural features at very fine length-scales, to the best of the authors’ knowledge, to date neither of the methods provides an outlook for large-scale cost-effective manufacturing of complex topologies.

The apparent stiffness of lattices when struts are in bending, is a trivial extension of the case when they are solid cylinders. We will not consider this case here. On the other hand, when these hollow struts are *c*ompressed diametrically, the deformation mechanism resembles that of a periodically pinched tube. The problem is further complicated by the fact that the pinching response may affect that at neighbouring lattice sites. Motivated by this, here we fabricate and theoretically characterise such porous material. In this study, first we present an extensive theoretical study on the mechanics of woodpile lattices with tubular cross-section struts subjected to compressive loading in the stacking direction. The analytical results are validated against numerical simulations. Furthermore, we propose a novel method to create hollow extrusions with controlled thickness and diameter using affordable FDM 3D printers. This is combined with the computer control provided by the commercial 3D printers so that extrusions could create structured matter to form 3D scaffolds. Woodpile structures with hollow struts are tested under compression. The results are compared with the theoretical analysis. The novelty is twofold. First, we derive, for the first time, the effective elastic modulus of periodic pinched-tube lattices using shell mechanics, yielding a closed-form relation in terms of relative density and thickness-to-radius ratio. This scaling extends beyond the simple Gibson-Ashby power laws, provides a practical design rule for tuning stiffness, and offers a foundation for analyzing other lattices that incorporate tubular elements. Second, we introduce an extrusion-based additive-manufacturing method that overcomes the usual constraint of simple cylindrical extrusion, enabling manufacture of lattices containing hollow tubes.

## Results

Understanding the deformation mechanisms of the woodpile structure with tubular cross-section struts is important in order to obtain the relationship between the apparent elasticity and porosity. Here we adapt the theory of elastic tubes under pinch load to the context and develop a theoretical model that provides the dependence of modulus on the geometric and material parameters. The key is the rapid decay away from the point of compression of the tube, so the effect of the deformation at the nearing sites can be neglected when the lattice spacing is large. The following analysis brings out the rapid decay, thus justifying this approximation. Also note that the decay is not monotonic; it is an oscillatory sinusoid.

**Fig. 1:**
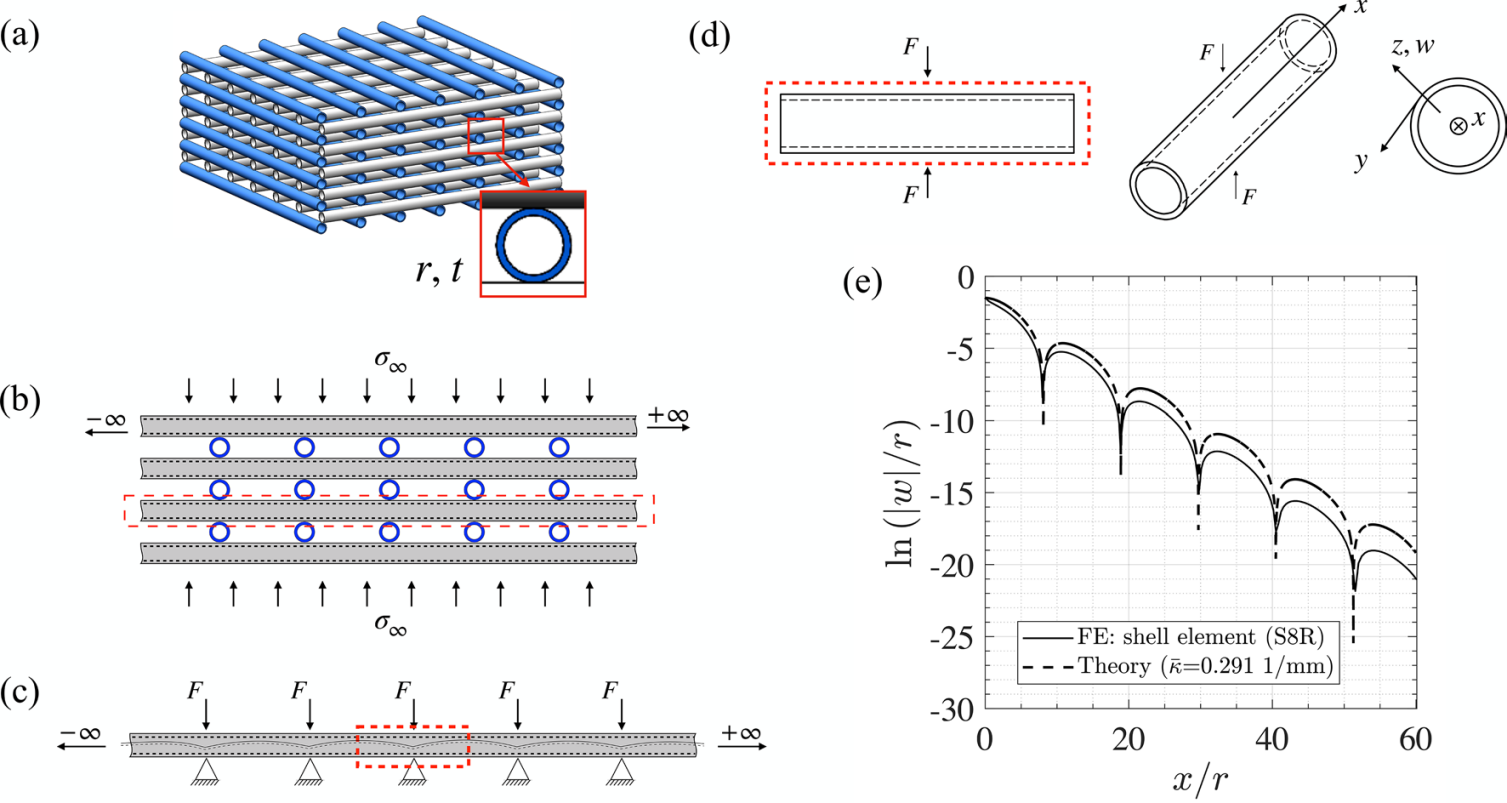
(**a**) Schematic of woodpile lattices with tubular struts, (**b**) 2D planar view of woodpile structure, (**c**) schematic of periodic pinch loads applied on a tubular filament in woodpile structure, (**d**) schematic of a long tube under pinching load, (**e**) radial deformation profile of a pinched tube along a line parallel to the cylinder axis (*w*/*r*) as a function of the scaled axial distance *x*/*r*, on a double-logarithmic scale, obtained from FE calculations and the developed theory. $\kappa$ found from FE, was used as a fitting parameter to generate the theoretical curve.

When a lattice with tubular struts is compressed, each hollow strut undergoes *p*eriodic diametrical compression, with a period equal to the lattice spacing Fig. 1a and b. Here we consider stacking arrangement in the form of a woodpile such that alternate layers are aligned one on top of another. The elastic response of each tube can be adequately modelled by periodically placed pinching (see, Fig. 1c).

### Structure-property relationship: scaling, persistence, and influence at neighbouring lattice sites

Consider an infinite elastic tube of radius *r* and thickness *t*, pinched diametrically by a force *F* applied at $x=0$, where *x* is the axial coordinate and *y* is the circumferential coordinate (see Fig. 1d). The governing equations of equilibrium, the so-called von Karman-Donnell equations, are a pair of coupled nonlinear partial differential equations in terms of two field variables: the deflection transverse to the cylindrical surface of the shell *w*(*x*, *y*), and the Airy’s stress function $\phi (x,y)$. Mahadevan et al.^29^, neglect nonlinear terms from the von Karman-Donnell equations, after seeking solutions of the form $w(x,y)=W(x)\cos (\pi y /2r)$ and $\phi (x,y)=\Phi (x) \cos (\pi y/2r)$. The function *W*(*x*) satisfies the following linear differential equation after making this approximation

1 $$\begin{aligned} W^{\prime \prime \prime \prime } + k^4 \approx 0, \end{aligned}$$

where a prime denotes differentiation with respect to *x*, the constant $k=\left[ \frac{t^2 \, \pi ^8}{12\, (1-\nu ^2)\, 2^8\, r^6}\right] ^{(1/4)}$, $\nu$ is the Poisson’s ratio of the material. Here *W*(*x*) is the amplitude of the separable solution that is modulated *y*-wise as per the function $\cos (\pi y/2r)$. The deformation profile along the line $y=0$ on the surface of the shell due to this force, which provides an interpretation for the function *W*(*x*), has the form $Wx)\sim e^{(-\kappa x)} \cos \,\left[ (\kappa x)+\alpha \right]$, for positive *x*, where $\kappa = k/\sqrt{2}$. The solution in^29^ has the correct functional form but the persistence length seems to be incorrect by a factor of ${\sqrt{2}}$, possibly due to an incorrect solution of the ordinary differential equation (1): it is of the form $W(x) \sim e^{-\kappa x}(\sin \kappa x + \cos \kappa x)$, rather than $W(x) \sim e^{-k x}(\sin k x + \cos kx)$. This will be discussed in detail elsewhere. The phase $\alpha$ depends on the boundary conditions. For an infinite cylinder, pinched in the middle, symmetry requires zero *x*-wise slope at the point of application of the load, i.e. $W^{\prime }(0)=0$, so that

2 $$\begin{aligned} W(x)=W_{0}e^{-\kappa x}(\sin \kappa x + \cos \kappa x) = {\sqrt{2}} W_{0} \cos (\kappa x+ \alpha ), \end{aligned}$$

with $\alpha = -\pi /4$. The displacement at the point of application of load is given by $W(0) = W_{0}$. An independent estimate for $W_{0}$ can be incorporated, for example, by using Calladine^30^ who provides an expression for *W*(0), using the kinematic relation between surface strains and change in Gaussian curvature for a *l*ong pinched tube that $W(0)=\frac{1}{1.23} \frac{F\,(1-{\nu }^2)}{Er}\left( \frac{r}{t}\right) ^{2.5}$, where *E* is Young’s modulus. This response has one length scale associated with it, which scales with the reciprocal of *k*. Note that the length scale of the oscillatory part is the same as that of spatial decay. It is the length scale at which tube recovers its undeformed shape; we will refer to this as the *persistence length* $\ell _p$. The persistence of a localized pinch in an elastic tube is characterised by a decaying oscillatory exponential with a persistence length given by $\ell _p=({2 \pi }/{\kappa })$^29,31^ (NB The difference between $\kappa$ and *k* as mentioned in the previous footnote).

Combining this with the expression for $W_0$^31^, we have

3 $$\begin{aligned} W(x)= \frac{1}{1.23} \frac{F\,(1-{\nu }^2)}{Er}\left( \frac{r}{t}\right) ^{2.5}e^{-\kappa x}(\sin \kappa x + \cos \kappa x). \end{aligned}$$

The above equation describes the axial profile of an infinitely long elastic cylinder pinched at $x=0$.

Because of the presence of the $e^{-\kappa x}$ term, we expect the contribution from the diametrical pinching of far off sites to rapidly decay. Nevertheless, we are in a position to express the response under one of the forces *F* as a sum of diametrical responses due to all the pinching forces at all other neighbouring sites. For linear elastic analysis, the radial displacement is given by superposition of responses due to all forces, i.e., $\delta = \delta _0 +\delta _+ + \delta _-$, where $\delta _0 = W_0$, i.e., diametrical compression in an infinite tube at the point of application of the force *F*, $\delta _+$ is the summed up response due to all forces that act in the domain $x\ge 0$, and $\delta _-$ is the summed up response due to all the forces in the domain $x\le 0$. For an infinite lattice, there exists symmetry at $x=0$, so that $\delta _+ = \delta _-$, hence $\delta = \delta _0 + 2\delta _+$. If the lattice spacing is $\lambda$, then $\delta$ can be expressed as an infinite sum

4 $$\begin{aligned} \delta = \frac{1}{1.23} \frac{F\,(1-{\nu }^2)}{Er}\left( \frac{r}{t}\right) ^{2.5} \left[ 1+ 2\sum _{p=1}^\infty e^{-p\kappa \lambda }(\sin p\kappa \lambda + \cos p\kappa \lambda ) \right] , \end{aligned}$$

because periodically placed forces are located at $x=0, \pm \lambda , \pm 2\lambda , \pm 3\lambda , \ldots$. The above expression brings out two things: (i) the diametrical compression in a periodically pinched tube scales as $\delta \sim \frac{F\,(1-{\nu }^2)}{Er}\left( \frac{r}{t}\right) ^{2.5} f( \lambda )$, and (ii) the effect of neighbouring sites rapidly decays away, hence for lattice spacing greater than the persistence length, as a first approximation, we could approximate the diametrical compression as $\delta \approx \frac{F\,(1-{\nu }^2)}{1.23 Er}\left( \frac{r}{t}\right) ^{2.5}$. For dense lattices, this is not valid and we restrict ourselves to the case when the lattice has low density (lattice spacing large compared to the persistence length) while developing the structure-property relationship later and also for our experimental work. The dependence of the apparent modulus of elasticity on the lattice geometry and the structural parameters of each hollow strut will be developed in the next section—while the scaling thus derived is correct for thin tubular structures, the accuracy of the proportionality constant will depend on lattice spacing.

The decaying sinusoid *W*(*x*) is described by—in addition to the amplitude $W_{0}$—just one parameter $\kappa$, which appears in both the decay rate and the oscillatory length scale. Sharp dips are expected in the logarithm of $\vert W(x)\vert$ at the zeros of the function *W*(*x*) given by $e^{-\kappa x}(\sin \kappa x+ \cos \kappa x) = 0$, i.e., at $\kappa x_{i} = (3\pi /4), (7\pi /4), (11\pi /4), \ldots$ Thus the location of these dips must have the ratio $x_{1}: x_{2}: x_{3} \ldots = 3:7:11: \ldots$ The functional form of *W*(*x*) is consistent with that of Eq. (2), however, the decay rate and the period—both related to the single parameter $\kappa$—are incorrectly estimated (as mentioned in the footnote). Therefore, we extract $\kappa$ the parameter from the location of the first two dips of finite element computations using $\overline{\kappa }= \pi /(\overline{x}_{2} - \overline{x}_{1} )$, where the overline represents estimated values from finite element calculations. A comparison is shown in Fig. 1e. The computationally obtained profile matches extremely well with the theoretical expectation of the location of dips as well as the slope of the exponential roll-off after fitting this parameter, thus confirming the correctness of the functional form in Eq. (2).

### FE simulation vs the analytical modulus-geometry-porosity relationship

**Fig. 2:**
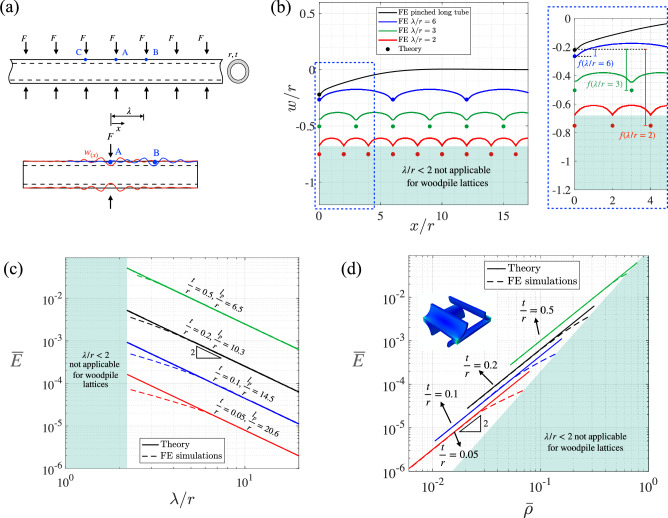
(**a**) A tube under periodic pinch load. The forces are $\lambda$ apart, (**b**) deformation profile of a periodically loaded tube for different values of $\lambda /r$ obtained from FE calculations (lines) and the developed theoretical deformation value for each case (points). The deformation under the applied forces is compared with the theoretical solution proposed. Deviation of deformation from $w_0$ are also shown as $f(\lambda )$ for various values of $\lambda$. (**c**) Relative modulus versus $\lambda /r$ for different *t*/*r* values. A comparison between theory and FE simulations shows an excellent agreement for large value of filament spacing. FE curves depart from theory when spacing between filaments $\lambda /r \approx 0.35 \, \ell _p/r$. (**d**) Relative modulus versus relative density for different *t*/*r* values. A comparison between theory and FE simulations shows an excellent agreement for small relative densities. For a constant value of relative density, relative modulus increases as the ratio *t*/*r* increases. The shaded region shows unachievable relative density with tubular woodpile lattices.

The deformation profile of a periodically pinched tube, Fig. 2a, with $t/r=0.05$ and $\lambda /r$=2, 3, and 6 obtained from FE simulations are shown in Fig. 2b. The radial displacement at the point of application of load can be obtained in terms of a function $f(\lambda )=\delta -W_0$, for various $\lambda /r$ values, are also shown in Fig. 2b.

In the context of woodpile lattices, for smaller filament spacing ($\lambda < \frac{\ell _p}{2\pi }$), the term $\sum _{p=1}^\infty e^{-p\kappa \lambda }(\sin p\kappa \lambda + \cos p\kappa \lambda )$ has a finite value depending on the *r*/*t* ratio, and $\lambda$. In other words, in a woodpile lattice with tubular struts when the spacing between filaments is smaller than persistence length of the filament, the effect of periodic force cannot be ignored. Eq. (4) provides us the apparent stiffness value for each case. To carry out this exercise, we focused on the case of periodic force of $F=0.4$ N, on a tube with $r= 1$ mm and $t=0.05$ mm, with filament spacing $\lambda = 2, 3,$ and 6 mm, respectively. In these cases, $f(\lambda )$ are found via theoretical calculations Eq. (4) and the results are shown as circular points on Fig. 2b for each case of $\lambda$. For $\lambda =6$ mm, the sum value is 0.10, and the theoretical results match FE simulations with less than 1$\%$ error. For $\lambda =3$ mm and 2 mm, the sum value is 0.54, and 1.21, respectively. These theoretical results lead to 5% and 9% error compared to FE simulations. These results demonstrate the effectiveness of the Eq. (4) for moderate spacing.

Figure 2c shows the apparent modulus versus $\lambda /r$ for different values of *t*/*r* comparing Eq. (5) with FE simulation results. Solid lines shows the theoretical trend, which corresponds to the power law mentioned above, for four different *t*/*r* values while broken lines shows the corresponding FE simulation results. There is an excellent agreement between the scaling theory and FE simulations for large value of filament spacing. Curves representing trends obtained from FE calculations depart from theory when $\lambda /r \approx 0.35 \, \ell _p/r$. This is due to the effect of additional response caused by forces from neighboring filaments when placed closely. Departure from theory is more pronounced for smaller filament spacing and lower values of *t*/*r*. The shaded area shows the unachievable filament spacing in tubular woodpile lattices.

In Eq. (4), if the lattice spacing is large, i.e., $\lambda \gg \frac{\ell _p}{2\pi }$ then $e^{- \kappa \lambda } \rightarrow 0$, so the second term in the right bracket becomes zero, hence $\delta =W_0$, which means $f(\lambda ) \rightarrow 0$. In other words, in a woodpile lattice with tubular struts when the spacing between filaments is larger than persistence length of the filament, the effect of periodic force can be ignored and apparent modulus can be found that is independent of $\lambda$. The *r*elative density-modulus relationship requires developing expressions for the *a*pparent stress ($\frac{F}{\lambda ^2}$ for a unit cell) and the *a*pparent strain ($=\frac{\Delta }{4r}$ for a unit cell), while incorporating the relevant lattice micromechanics. The crucial part of this is determination of the response of hollow elastic structures when compressed diametrically and finding the diametrical compression deformation $W_0 \; (=\Delta /4$ where $\Delta$ is total deformation of unit cell). The ratio of the apparent stress and the apparent strain (both nominal in the small strain limit) provides an expression for the apparent Young’s modulus

5 $$\begin{aligned} \left<E\right> =\frac{\left<\sigma \right>}{\left<\varepsilon \right>}= \frac{1.23}{1-\nu ^2}\,\frac{t^\frac{5}{2}}{\lambda ^2\, r^{\frac{1}{2}}} \, E. \end{aligned}$$

This effectively assumes no response due to compressive forces at neighbouring locations. Hence, for a constant *t*/*r* value, the apparent modulus follows an inverse-second power law with $\lambda /r$.

The relative apparent density of this structure can be obtained as ${\overline{\rho }}=\frac{\pi }{2} \frac{(r_o^2-r_i^2)}{r_o\lambda }$($=\frac{\pi t}{\lambda }$ for thin tube), where $r_o$ and $r_i$ are the outer and inner diameter of the tubular struts. For Poisson’s ratio equal to $\nu = 0.36$, which is realistic for the polymeric material used for experimental work later, and for $\overline{\rho } = \pi t/\lambda$, the expression found in Eq. (5) can be rewritten in terms of the relative apparent density as:

6 $$\begin{aligned} \left<E\right>= 0.143 \, (\overline{\rho }) ^2 {\sqrt{\frac{t}{r}}} \, E. \end{aligned}$$

Note that the above expression is not a simple power law scaling with the relative density, unlike that obtained for lattices with solid filaments^32^. The relationship between the apparent modulus and relative density follows a second power law for a *c*onstant value of *t*/*r*. The effect of apparent density is investigated by keeping *t*/*r* constant. A comparison between theoretical trend (solid lines) and FE simulation (broken lines) for apparent modulus versus relative density for different *t*/*r* values is shown in Fig. 2d. For small relative densities, the results of FE simulations are in excellent agreement with theoretical prediction. Curves obtained from FE calculations depart from theory due to the effect of persistence caused by forces from neighbouring filaments. For a constant value of relative density, the relative modulus increases as the ratio *t*/*r* increases (Eq. (6)). The shaded region shows unachievable relative density with tubular woodpile lattices. The results of this section can be used to assess the effect of relative density and *t*/*r* ratio on stiffness of these lattices.

Rearranging terms in eq. (6), a more general relationship connecting the scaled apparent modulus $\overline{E}$, the ratio of the tube radius to the thickness (*r*/*t*) and the scaled apparent density $\overline{\rho }$, is obtained as

7 $$\begin{aligned} \overline{E} \left( \frac{r}{t}\right) ^{\frac{1}{2}} \sim (\overline{\rho })^{2}, \end{aligned}$$

where the relative apparent modulus $\overline{E} = \langle E\rangle / E$, and the relative apparent density $\overline{\rho }= \langle \rho \rangle / \rho$, where $\langle \rho \rangle$ is the apparent density of the lattice material, and $\rho$ is the density of the parent material of the tubes. Eq. (7) highlights the absence of frequently used power laws in the context of porous and lattice materials of the form $\overline{E}\sim (\overline{\rho })^{m}$, where *m* is some exponent, for this class of materials and thus emphasizes the inadequacy of such a power law for this lattice architecture. We validate the structure-property relationship (7) experimentally by taking up an experimental campaign with several samples of varying tube diameter and lattice spacing. This will be taken up next.

### Experimental results, validation and discussions

**Fig. 3:**
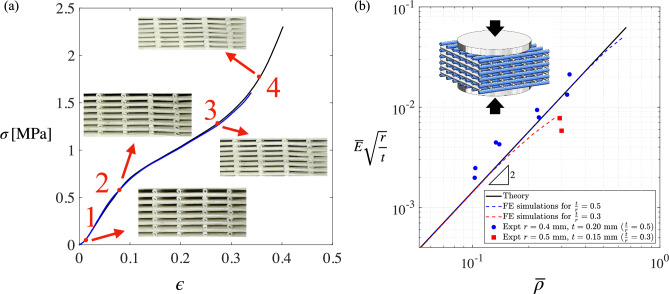
(**a**) A typical stress-strain behaviour of tubular-strut woodpile lattice (3D printed samples fabricated by nozzles with $d_o=1.0$ mm and $d_i=0.6$ mm; spacing 5.0 mm) under compression. (**b**) A comparison between theoretically predicted power law relationship, and the experimentally obtained measured $\frac{\left<E\right>}{E}{\sqrt{\frac{r}{t}}}$ versus apparent density relationship on a logarithmic scale. The slope 2 confirms the relationship $(\langle E \rangle / E)(r/t)^{\frac{1}{2}} \sim (\overline{\rho })^{2}$.

After evaluating several manufacturing methods, ceramic 3D printing was chosen to manufacture the bespoke dies required for the 3D printer. Details of the nozzle manufacturing process are explained in the author’s PhD thesis^33^. The woodpile samples were subsequently fabricated and tested in the lab. The details are explained in the Methods section. The experimental results and comparison with computations and theoretical predictions are presented in Fig. 3.

Typical stress-strain curves obtained from experiments are presented in Fig. 3a. The apparent stress and the apparent strain are calculated from the load and the projected area of the overall sample, as well as end-to-end deformation over the original sample length. These values represent nominal *a*pparent stress and *a*pparent strain rather than true stress/true strain. The response is non-linear when the strain becomes large. There are four stages of deformation that can be identified: (1) localised tube squeeze, (2) yielding of the tubular segments followed by non-linear response, (3) plastic deformation, (4) contact and densification of ovalized tubular layers. These phases are shown in the figure using four key points, as labelled. While the response for large deformation is clearly non-linear, we restrict out attention to the *l*inear part where the strain is small, as is customary in assessing moduli of elasticity. The slope of the *i*nitial linear part, in the interval labelled points 1 and 2, is used for determining the apparent Young’s modulus $\langle E \rangle$. Here we are not concerned with the apparent properties in the nonlinear regime, hence response in large strain region is not used for further analysis and interpretation. For each specimen, the apparent density was calculated by weighting samples before the test and dividing it by its volume based on its external shape. Thus, from samples of different lattice spacing $\lambda$ and filament mean radius *r* and thickness *t*, the relationship between the volume fraction, the apparent modulus and *t*/*r* ratio can be experimentally determined. Since the apparent modulus is not a function of the apparent density alone, the micromechanics suggests a natural grouping of non-dimensional term $\frac{\langle E \rangle }{E}{\sqrt{\frac{r}{t}}}$, which scales with the apparent density according to $\left( \overline{\rho }\right) ^2$ as per Eq. (6).

Figure 3b shows a comparison between theoretically predicted power law relationship, and the experimentally obtained measured modulus versus apparent density relationship. Experimental data for two different nozzle diameter are overlaid on to a single graph. The results fall closely along a line of slope 2 on a log-log plot, as predicted by the analysis. This confirms the approximate analytical relationship $\bar{E}\sqrt {r/t} \propto ( {\overline{\rho }})^{2}$. The differences can be attributed to factors such as: (i) manufacturing tolerances in the fabrication of samples, (ii) experimental errors, (iii) Finite-thickness of tubular members rather than theoretically thin tubes. Our plots show relative stiffness versus each specimen’s experimentally measured density. Due to unavoidable manufacturing tolerances, no two specimens share exactly the same density, so we do not have replicated points at a single relative density on which to place vertical error bars. Hence we present the full cloud of data, which reflects both measurement noise and real variations.

The present study makes several simplifications and assumptions in order to obtain a relationship between the apparent Young’s modulus and the geometric and material parameters of the lattice. Due to these simplifications, there are limitations: (i) The analysis is limited to linear elastic range, as required by the definition of apparent elastic moduli, (ii) The influence of compression from neighbouring sites has been neglected in view of the rapidly decaying exponential term—thus this study is valid for lattice spacing much larger than the radius, (iii) results make use of thin shell theory, which means the thickness of the tube is assumed to be much smaller than the diameter, (iv) The lattice is assumed to be infinite, whereas real structured matter is of finite spatial extent. The validity requires the overall dimensions much larger than the characteristic lattice dimension, i.e., the lattice spacing. For moderate lattice spacing, the effect of neighbouring sites can still be included effectively, although a simple modulus-geometry-porosity relationship such as that in eq (7) is not applicable.

Despite these limitations, we have obtained the analytical relationship $\overline{E} {\sqrt{r/t}} \sim (\overline{\rho })^{2}$ and have been able to demonstrate the validity of this relationship computationally and experimentally. This also highlights that a power law—directly between the apparent modulus and the volume fraction—does not exist for this lattice architecture and the relevant structure-property relationship requires the $(r/t)^{\frac{1}{2}}$ term. Experimentally observed trend is in line with this relationship.

## Conclusions

This paper explores the mechanics of woodpile lattices with tubular struts under stress in the stacking direction leading to the diametrical compression of hollow tubes—analytically, numerically, and experimentally. Recently, there has been significant interest in replacing solid struts with hollow ones, particularly in biomedical applications, due to their ability to enhance vascularization and porosity. This modification also provides advantages in other fields by reducing the overall material weight. Hollow struts significantly alter the mechanical properties of lattices, mainly by reducing their density, while also offering greater flexibility in material design through an additional geometric parameter—the hollowness of the struts.

The study investigates how different structural and material parameters, such as tube thickness, radius, and center-to-center spacing, etc., influence the mechanical response of these lattices. For the theoretical analysis, each hollow strut is modeled as a hollow shell subjected to diametrically opposite forces, applied periodically by the struts in adjacent layers. The behavior of an elastic tube under a pinching load is used to derive an expression for the apparent modulus, linking remote stress to apparent strain. The apparent modulus is found to depend on relative density, following a second power law, as well as on the square root of the ratio *t*/*r*. When the distance between struts is small relative to the persistence length, the influence of neighboring layers becomes significant. In these cases, the modulus-density relationship is examined numerically, confirming a deviation from the theoretical model.

We also presented a design and manufacturing method for FDM 3D printer dies capable of producing tubular filaments. These nozzles were successfully fabricated using ceramic 3D printing techniques. Lattices containing tubular elements with various combinations of radii and thicknesses were then produced. These lattices were mechanically tested under compression, and the results demonstrated excellent agreement with the theoretical predictions. The findings provide valuable insights for designing and manufacturing tubular woodpile lattices with programmable mechanical properties.

## Methods

### Design and manufacture of bespoke 3D printing dies and woodpile lattice 3D printing

**Fig. 4:**
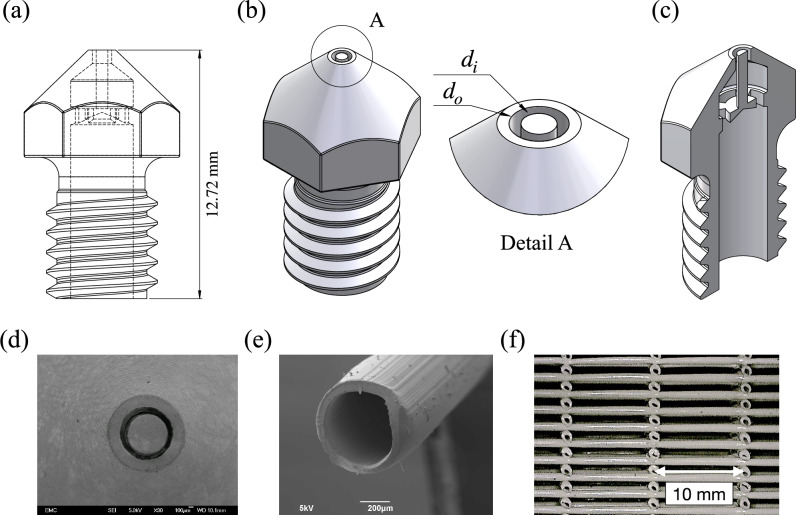
(**a**) CAD drawing design of nozzle with capability of printing tubular struts, (**b**) 3D image of the designed nozzle with magnified detail of the orifice, and (**c**) 3D image of the internal features in the customised nozzle, (**d**) Ceramic 3D printed customized nozzles with tubular shape material outlet. (**e**) A typical tubular strut extruded through one of the nozzle with $d_o=1.1$ mm and $d_i=0.8$ mm. (**f**) An optical image of the woodpile structure fabricated by nozzle with $d_o=1.0$ mm and $d_i=0.6$ mm, during compression testing. The large lattice spacing for a typical sample relative to the tube radius justifies ignoring effects from neighbouring lattice sites.

As mentioned earlier, 3D printing of tubular struts at sub-millimeter scale is intricate and cannot be achieved by using commercial 3D printer. In the approach taken here, the 3D printing nozzle is re-designed so that it can extrude tubular struts. After fabricating several prototypes using ceramic 3D printing, the idea of placing guiding core inside the traditional FDM 3D printing nozzle appeared most promising. However, it is a complex structure to fabricate economically, if not commercially done use large volume manufacturing methods. An advantage of ceramic parts produced by 3D printing using ceramic powders is their high resolution. Lithographic additive manufacturing technology (L-AMT) provided by International Syalons (Newcastle) Ltd., Wallsend, UK, is used here. The main strength of this fabrication method over other possible manufacturing route lies in the fact that objects containing internal features can be fabricated with high resolution. The nozzles were fabricated using Lithoz CeraFab 3D printing technology, then de-binded and sintered like conventionally manufactured ceramics. The material used in this technology is Syalon 101 which is the first of the Si-Al-O-N family of ceramic materials developed and characterised. This material is manufactured in controlled conditions and is suitable for applications that require high strength, toughness, and wear resistance. Figure 4a, b, c shows the internal structure and conceptual design of the nozzles carried out in Solidworks. Images of the outlets of the printed nozzles are shown in Fig. 4d. An SEM micrograph of a typical tubular strut extruded through one of the nozzles ($d_o=1$ mm, $d_i=0.7$ mm) is presented in Fig. 4e. The core is attached to the inner wall of the nozzle via three small bridges. The size and position of these bridges were optimised for better performance of these nozzles and through several iterative trials. Four different configurations of nozzles with tubular outlet were designed: (1) $d_o=1$ mm, $d_i=0.7$ mm, (2) $d_o=0.9$ mm, $d_i=0.5$ mm, (3) $d_o=1.2$ mm, $d_i=1$ mm, and (4) $d_o=2$ mm, $d_i=1.6$ mm.

Woodpile lattices were printed on an Ultimaker 2 Extended+ using our in-house G-code, which allows control of lattice spacing; interchangeable nozzles were used to vary filament diameter, see Fig. 4F. Process parameters were 190$\phantom{0}^{\circ }$C nozzle temperature, $800 \textrm{mm}/\textrm{min}$ print speed, and 80% fan. A 4% line overlap (relative to filament outer diameter) was used to ensure interlayer bonding with minimal geometric perturbation. As expected, extrudate geometry deviates slightly from the nominal nozzle size ($<9\%$) and exhibits mild ellipticity due to flattening during solidification (wider in-plane, shorter through-thickness). For prints with a 1 mm nozzle (nominal outer diameter), measured filament diameters were (1053 ± 33) µm and (926 ± 25) µm, in horizontal and vertical directions, respectively. The scatter reflects normal process variability, whereas the horizontal-vertical mean difference is a systematic effect of bead flattening. Relative density was computed from specimen mass, bulk material density, and external specimen volume. Compressive tests followed ASTM D1621: specimens were 50 mm $\times$ 50 mm in plan and $\sim$ 25 mm in height, loaded uniaxially on an Instron 5569 at a strain rate of $3\times 10^{-4}~\textrm{s}^{-1}$, with in-situ imaging using a Dino-Lite microscope. Samples with different filament spacing were fabricated and tested.

### Finite element simulations

The accuracy of the approximate analytical expressions for deformations was checked against FE calculations performed using the commercial finite element code ABAQUS/CAE/Standard 6.18$^{\mathrm {\text{\textregistered} {}}}$ (Simulia, Dassault Systems, Providence, RI, USA). The material was assumed to be linear elastic material with Young’s modulus $E=2290$ MPa and Poisson’s ratio $\nu =0.36$. Due to symmetry, half of the unit cell of a woodpile structure is modelled in ABAQUS in order to evaluate results from analysis and laboratory experiments. Filaments in lattices are modelled as elastic tubes and meshed using C3D10 elements that use quadratic interpolation. The diameter of the tubes is taken as $r=1$ mm, center-to-center separation between the filaments $\lambda$ and thickness *t* were varied systematically to obtain a range of porosity, in order to study their effect upon the apparent stiffness. These dimensions are chosen in the range of extrusions from the customized FDM nozzles. Constant vertical displacement is applied to the top part of the model. The nodes on the bottom surface are constrained in the $y$-direction. Due to the periodicity and symmetry of the structure, all the nodes lying on the vertical surfaces are constrained to remain in their plane, allowing displacements in that plane. Reaction forces are calculated at the bottom surface by summing up all the nodal reactions. Using the dimensions of the model and the obtained reaction force corresponding to the applied displacement, the apparent lattice stiffness is calculated. The bonding between the filaments was modelled by considering a 0.001 mm overlap between stacking layers. Actual apparent density $\overline{\rho }$ is calculated from ABAQUS using mass property calculation tool.

## Data Availability

Data are available upon request to the corresponding author (Dr. Faezeh Shalchy, f.shalchy@hw.ac.uk).
